# Chlorogenic acid inhibits intestinal lipid uptake and promotes adipose lipid catabolism via epithelium-derived exosomes in ob/ob mice

**DOI:** 10.3389/fphys.2026.1896020

**Published:** 2026-07-29

**Authors:** Yongwang Yan, Yifang Zhou, Di Wang, Xu Zhou

**Affiliations:** 1 Pharmaceutical College, Changsha Health Vocational College, Changsha, China; 2 Hunan Provincial Hospital of Integrated Traditional Chinese and Western Medicine (The Affiliated Hospital of Hunan Academy of Traditional Chinese Medicine), Changsha, China

**Keywords:** chlorogenic acid, exosomes, intestine, lipid metabolism, obesity

## Abstract

Chlorogenic acid has been widely reported to regulate lipid metabolism and alleviate obesity; however, its effects on intestinal lipid uptake and adipose tissue catabolism, as well as the underlying mechanisms, remain incompletely understood. In the present study, diabetic ob/ob mice were treated with chlorogenic acid, which significantly improved metabolic parameters, including body weight, serum triglyceride levels, glucose tolerance, and insulin sensitivity. Notably, chlorogenic acid reduced intestinal lipid content and downregulated the expression of genes involved in lipid transportation. Concurrently, epididymal adipose tissue weight was decreased, accompanied by enhanced expression of genes associated with lipid catabolism. Mechanistically, chlorogenic acid decreased triglyceride level and suppressed PPARγ protein expression in IPEC-J2 cells. Activation of PPARγ attenuated the inhibitory effects of chlorogenic acid on lipid accumulation, indicating a critical role of PPARγ signaling. In addition, chlorogenic acid altered the concentration of epithelial cell–derived exosomes. These exosomes reduced triglyceride levels and modulated the expression of lipid metabolism–related genes in 3T3-L1 cells. Collectively, our findings suggest that chlorogenic acid may exert metabolic benefits, at least in part, through a coordinated intestine–adipose axis and provide preliminary evidence supporting a potential role for exosome-mediated inter-organ communication in the regulation of lipid homeostasis.

## 1 Introduction

Chlorogenic acid, one of the most abundant plant-derived phenolic acids, has attracted considerable attention due to its diverse biological activities, including antioxidant, anti-inflammatory, antimicrobial, anticancer, antidiabetic, and anti-obesity effects (Yan et al., 2020a; He et al., 2025b). Among these biological functions, the anti-obesity and antidiabetic properties of CGA have been increasingly linked to its ability to modulate lipid metabolism. Dysregulation of lipid metabolism is a key pathological feature underlying several metabolic disorders, including obesity, type 2 diabetes, nonalcoholic fatty liver disease, and cardiovascular diseases (Ziolkiewicz et al., 2024; He et al., 2025b). Accordingly, considerable efforts have been devoted to elucidating the mechanisms by which CGA regulates lipid metabolism and exerts metabolic benefits.

Chlorogenic acid has been shown to improve systemic lipid metabolism by promoting the clearance of triglycerides, total cholesterol, and free fatty acids, as well as enhancing glucose tolerance and insulin sensitivity (Ma et al., 2015; Yan et al., 2020b). In addition, chlorogenic acid alleviates obesity in multiple rodent models, including high-fat diet (HFD)-induced obese mice and diabetic db/db mice (Wang et al., 2019; Yan et al., 2022). Most previous studies have focused on hepatic lipid metabolism, demonstrating that chlorogenic acid inhibits the expression of genes involved in lipid synthesis while promoting those related to lipolysis and fatty acid oxidation (Wan et al., 2013; Yan et al., 2022). Beyond the liver, chlorogenic acid also exerts metabolic regulatory effects in other tissues. For instance, it enhances thermogenesis in brown adipocytes by improving mitochondrial function (Han et al., 2019), and reduces lipid accumulation and fibrosis in the kidney (Yang et al., 2024).

However, whether orally administered chlorogenic acid can regulate intestinal lipid metabolism remains largely unclear. Moreover, although chlorogenic acid has been reported to bind to the extracellular domain of the transmembrane receptor Notch1 and inhibit Notch signaling in the kidney (Yang et al., 2024), it is still unknown whether chlorogenic acid directly reaches target tissues such as adipose tissue via the circulatory system or exerts its effects through indirect mediators.

Recently, exosomes have emerged as critical mediators of intercellular communication among metabolic tissues. Evidence suggests that intestinal epithelial cell-derived exosomes can enter adipose tissue and promote thermogenesis (Han et al., 2025). In addition, intestinal exosomes may regulate adipocyte browning through their cargo, such as microRNAs (Di et al., 2025). Therefore, in the present study, we first investigated whether chlorogenic acid regulates intestinal lipid metabolism in diabetic ob/ob mice. Furthermore, we examined whether chlorogenic acid alters epithelium-derived exosomes and facilitates their transfer to adipose tissue, thereby mediating metabolic regulation.

## 2 Materials and methods

### 2.1 Animals and experimental design

Six-week-old male leptin-deficient ob/ob mice on a C57BL/6J background and age-matched wild-type (WT) C57BL/6J mice (specific pathogen-free grade) were obtained from Slac Laboratory (Changsha, China). All animals were housed under controlled conditions (22 ± 2 °C, 50 ± 5% humidity, 12 h light/12 h dark cycle) with free access to food and water. After one week of acclimatization, fourteen ob/ob mice were randomly divided into two groups: a chlorogenic acid-treated group (CHL) and an untreated model control group (OB). Seven WT mice were used as the normal control group (CON). Mice were orally gavaged once daily with either chlorogenic acid (0.25 g/kg body weight, dissolved in 0.1 mL PBS) or an equal volume of PBS. The chlorogenic acid dosage used in this study was selected according to our previously published studies (Yan et al., 2022). The experiment lasted for 8 weeks, during which body weight and feed intake were recorded regularly. All animal procedures were conducted in accordance with the guidelines of Hunan Academy of Traditional Chinese Medicine and were approved by the Institutional Animal Care and Use Committee of Hunan Academy of Traditional Chinese Medicine (SBWJW2024-0001).

### 2.2 Oral glucose tolerance test and insulin-assisted oral glucose tolerance test

One week before the end of the experiment, GTT and IA-OGTT were performed. For the GTT, mice were fasted for 6 h and orally gavaged with glucose at a dose of 2.0 g/kg body weight. For the insulin-assisted oral glucose challenge, mice were fasted for 6 h, orally gavaged with glucose at a dose of 2.0 g/kg body weight, and simultaneously intraperitoneally injected with insulin at a dose of 0.65 U/kg body weight. Blood samples were collected from the tail vein, and glucose concentrations were measured at 0, 15, 30, 60, and 120 min.

### 2.3 Triglycerides tolerance test

For the TG tolerance test, mice were fasted overnight and then orally gavaged with olive oil at a dose of 10 μL/g body weight as previously described (Hou et al., 2024). Blood samples were collected at 0, 1, 2, 3, and 4 h after gavage, and serum TG levels were subsequently measured.

### 2.4 Sample collection

Blood samples were firstly collected from the retro-orbital sinus, and serum was obtained after separation. Afterward, mice were euthanized by cervical dislocation, and liver, inguinal, and epididymal adipose tissues were excised and weighed. In addition, liver, jejunum, and ileum samples were collected and stored at −80 °C for subsequent analyses.

### 2.5 Serum biochemistry assay

The levels of glucose, total cholesterol (TC), and TG in serum were measured using commercial kits (Boyan, Nanjing, China) following the manufacturers’ instructions.

### 2.6 Determination of TG content in tissue

For the determination of TG content in liver, jejunum, and ileum tissues, approximately 20 mg of tissue was homogenized in 5% Triton X-100 in saline. The homogenates were then extracted with methanol:chloroform (1:2, v/v), shaken for 12 h, and subsequently dissolved in isopropanol containing 10% Triton X-100 in saline. TG concentrations were finally measured using commercial kits (Boyan, Nanjing, China) according to the manufacturer’s instructions.

### 2.7 RT‐qPCR analysis

Total RNA was extracted using TRIzol reagent (Invitrogen, Shanghai, China). Complementary DNA was synthesized using a Reverse Transcription Reagent Kit (Thermo Fisher Scientific, Shanghai, China). Quantitative real-time PCR (RT–qPCR) was performed using an SYBR Green PCR Kit (Thermo Fisher Scientific) according to the manufacturer’s instructions. Relative gene expression levels were calculated using the 2^−ΔΔCt^ method, with β-actin used as an internal control. Primer sequences are listed in **Supplementary Table 1**.

### 2.8 Western blotting analysis

Tissue samples and cells were lysed, and protein concentrations were determined using a bicinchoninic acid (BCA) assay kit (Beyotime, Shanghai, China). Equal amounts of protein were subjected to SDS–PAGE and subsequently transferred onto polyvinylidene fluoride membranes (Bio-Rad, CA, USA). The membranes were blocked with bovine serum albumin and then incubated sequentially with primary antibodies (PPARγ, CD9, TSG1010; Abcam, Shanghai, China) and appropriate secondary antibodies. Protein bands were visualized using an enhanced chemiluminescence detection system. Protein expression levels were normalized to GAPDH.

### 2.9 Chlorogenic acid treatment on IPEC-J2 cells

IPEC-J2 cells were cultured in Dulbecco’s Modified Eagle Medium (DMEM) supplemented with 10% fetal bovine serum and 1% penicillin–streptomycin under standard conditions (37 °C, 5% CO_2_). IPEC-J2 cells were obtained from Pricella Biotechnology Co., Ltd. (Wuhan, China) and were originally sourced from DSMZ (Braunschweig, Germany). Cells were routinely tested and confirmed to be free of mycoplasma contamination, and experiments were conducted using cells between passages 25 and 40. Upon reaching approximately 70–80% confluence, cells were treated with palmitic acid (PA, 50 μM) in the presence or absence of 30 μM chlorogenic acid and 1 μM rosiglitazone (an agonist of PPARγ) for 24 h. PA was prepared as a stock solution conjugated with bovine serum albumin (BSA) to enhance solubility. Control cells were treated with an equivalent volume of vehicle. After treatment, cells were collected for subsequent analyses.

### 2.10 Chlorogenic acid treatment on intestinal organoids

Intestinal crypts were isolated as previously described (Sato et al., 2009). Briefly, the small intestine was opened longitudinally, rinsed with DPBS, and cut into 0.5–1.0 cm fragments. Tissues were incubated in dissociation buffer containing 2.0 mM EDTA and 1.0 mg/mL collagenase II at 37 °C for 20 min. The released crypts were filtered through a 70 μm strainer and collected by centrifugation at 500 g for 10 min. Crypts were resuspended in culture medium, mixed with Matrigel, and seeded into pre-warmed 96-well plates. After polymerization, complete medium was added and organoids were maintained at 37 °C with 5% CO_2_, and the medium was changed every 2 days. Then, organoids were treated with 50 μM PA, in the presence or absence of 30 μM chlorogenic acid and 1 μM rosiglitazone for 24 h.

### 2.11 Immunofluorescence staining

Intestinal organoids were fixed with 4% paraformaldehyde for 20 min at 22 ± 3 °C, followed by permeabilization with 0.2% Triton X-100 for 10 min. Samples were then blocked with 5% BSA and incubated overnight at 4 °C with primary antibodies (PPARγ, CD36; Abcam) diluted in 5% BSA. After washing, the samples were incubated with appropriate fluorescent secondary antibodies, and nuclei were stained with 4′,6-diamidino-2-phenylindole (DAPI; Beyotime). Fluorescence images were captured using a laser scanning confocal microscope (LSCM; Zeiss, Oberkochen, Germany). The mean fluorescence intensity of each image was quantified using ImageJ software under identical acquisition and analysis settings.

### 2.12 Isolation and characterization of exosomes from IPEC-J2 cells

The culture medium of IPEC-J2 cells was collected and subjected to sequential centrifugation at 1,000 *g*, 3,000 *g*, and 10,000 *g* for 30 min at 4 °C to remove cells, debris, and large vesicles. The resulting supernatant was filtered through a 0.22 μm membrane to eliminate large particles, followed by ultracentrifugation at 150,000 *g* for 3 h at 4 °C (Beckman Coulter Optima L-100XP). The exosomes pellet was resuspended in 100 μL PBS. Exosomes morphology was observed by transmission electron microscopy (TEM), and the size distribution and concentration were determined using nanoparticle tracking analysis (NTA; NanoFCM N30E, Shanghai, China). Protein concentration was quantified using an BCA protein assay kit (Beyotime). The particle-to-protein ratio ranged from 6.3 × 10^9^ to 7.5 × 10^9^ particles per μg protein.

### 2.13 Exosomes treatment on 3T3-L1 cells

3T3-L1 preadipocytes were induced to differentiate into mature adipocytes and then were treated with 50 μg exosomes derived from IPEC-J2 cells or from IPEC-J2 cells treated with 30 μM chlorogenic acid. An equal volume of culture medium processed in parallel without IPEC-J2 cells were used as control treatment. After 24 h of treatment, cells were harvested for subsequent analyses.

### 2.14 Statistical analysis

Data are presented as mean ± standard error of the mean (SEM). Statistical analyses were performed using GraphPad Prism 8.0 software. Differences among groups were analyzed by one-way analysis of variance (ANOVA) followed by the Student–Newman–Keuls *post hoc* test. A value of *P* < 0.05 was considered statistically significant. Statistical significance in figures is indicated as *P < 0.05, **P < 0.01, ***P < 0.001, and ****P < 0.0001.

## 3 Results

### 3.1 Chlorogenic acid alleviates diabetic symptoms in ob/ob mice

**Figure 1:**
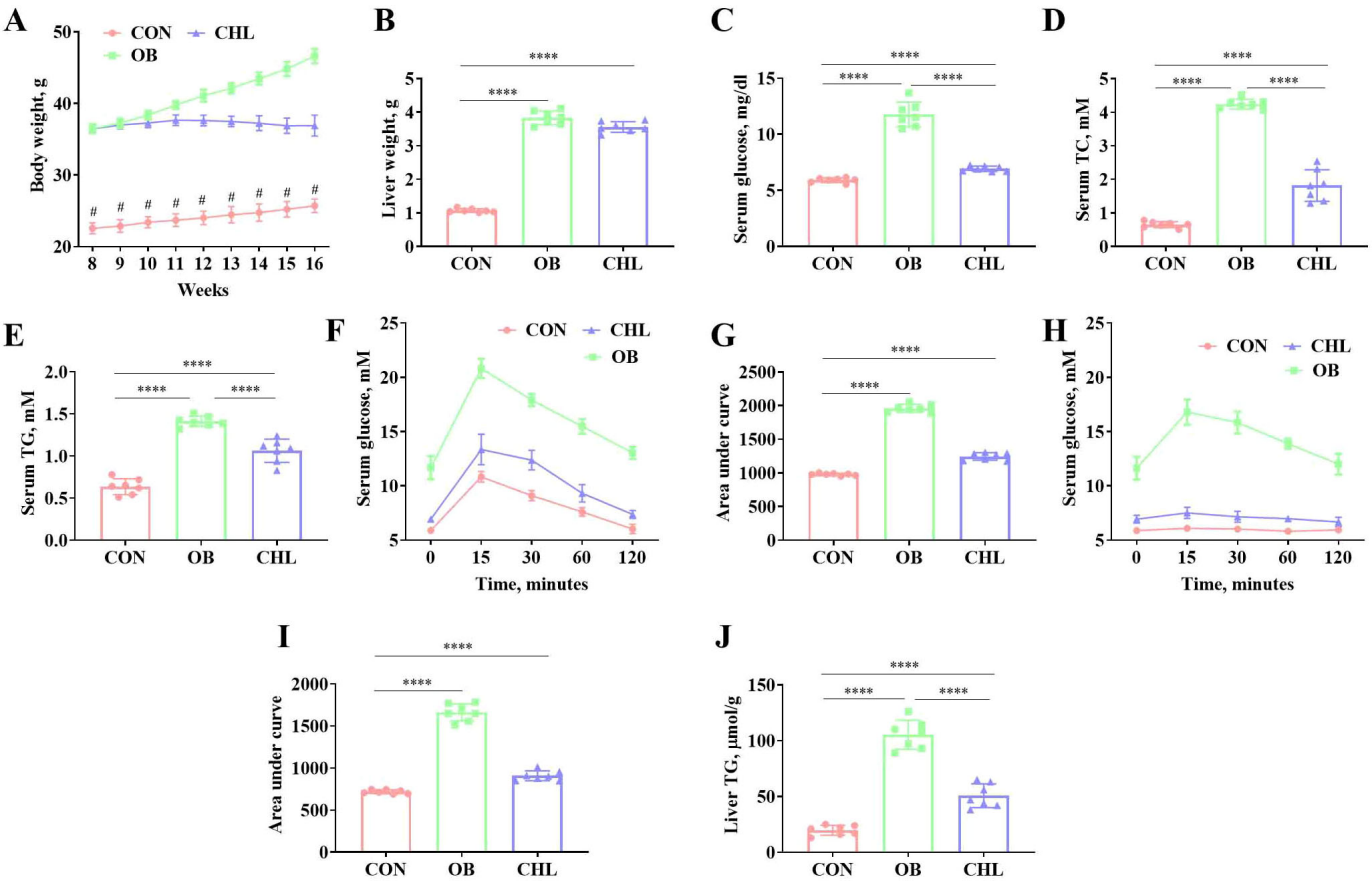
Chlorogenic acid alleviates diabetic symptoms in ob/ob mice. **(A)** Body weight; **(B)** Liver weight; **(C)** Serum glucose level; **(D)** Serum TC level; **(E)** Serum TG level; **(F)** Glucose tolerance test (GTT); **(G)** Area under curve of GTT; **(H)** Insulin-assisted oral glucose tolerance test (IA-OGTT); **(I)** Area under curve of IA-OGTT; **(J)** Liver TG level. TC, total cholesterol; TG, Triglycerides. CON, control WT mice; OB, control ob/ob mice; CHL, ob/ob mice treated with chlorogenic acid. Data were expressed as mean ± SEM. n = 7. ^*^P<0.05, ^**^P<0.01, ^***^P<0.001, ^****^P<0.0001.

We first evaluated the effects of chlorogenic acid in diabetic ob/ob mice. After 8 weeks of treatment, chlorogenic acid significantly reduced body weight (**Figure 1A**), and liver weight was decreased although it did not reach significance compared with diabetic ob/ob mice (**Figure 1B**). In parallel, serum biochemical analysis showed that chlorogenic acid significantly decreased the levels of glucose (**Figure 1C**), total cholesterol (TC) (**Figure 1D**), and TG (**Figure 1E**). Moreover, GTT and ITT results demonstrated that chlorogenic acid improved both glucose tolerance (**Figures 1F, G**) and insulin sensitivity (**Figures 1H, I**). Consistently, hepatic TG content was also significantly reduced following chlorogenic acid treatment (**Figure 1J**).

### 3.2 Chlorogenic acid decreases intestinal lipid uptake in ob/ob mice

**Figure 2:**
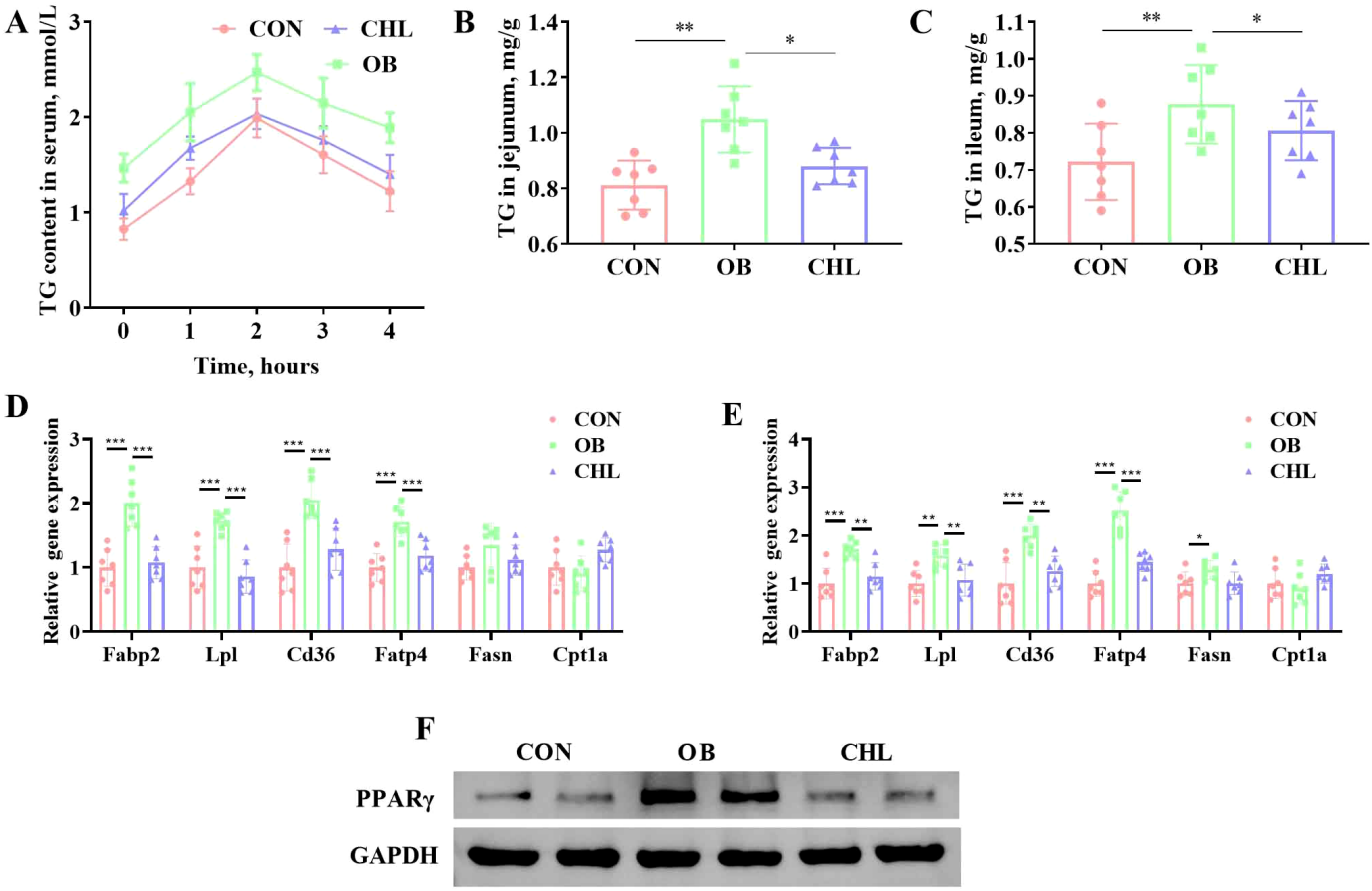
Chlorogenic acid decreases intestinal lipid uptake in ob/ob mice. **(A)** TG tolerance test; **(B)** TG content in the jejunal tissue; **(C)** TG content in the ileal tissue; **(D)** Relative gene expression in the jejunal tissue; **(E)** Relative gene expression in the ileal tissue; **(F)** PPARγ protein expression in the ileal tissue. Data were expressed as mean ± SEM. n = 7. ^*^P<0.05, ^**^P<0.01, ^***^P<0.001.

We next investigated the effects of chlorogenic acid on intestinal lipid metabolism. The TG tolerance test demonstrated that chlorogenic acid significantly reduced lipid uptake in ob/ob mice (**Figure 2A**). Consistently, TG levels in both the jejunum and ileum were significantly decreased following chlorogenic acid treatment (**Figures 2B, C**), further supporting reduced intestinal lipid absorption. To explore the underlying mechanisms, we analyzed the expression of genes involved in lipid transport and metabolism. Chlorogenic acid significantly downregulated the expression of lipid transport–related genes, including *Fabp2*, *Lpl*, *Cd36*, and *Fatp4*, in both the jejunum and ileum (**Figures 2D, E**). In contrast, chlorogenic acid selectively reduced the expression of the lipogenic gene *Fasn* in the ileum but not in the jejunum, and had no significant effect on the expression of the fatty acid oxidation–related gene *Cpt1a*. Furthermore, we examined the protein expression of PPARγ, a key regulator of lipid metabolism, and found that its expression in the ileum was significantly decreased following chlorogenic acid treatment (**Figure 2G**). These findings indicate that the inhibitory effect of chlorogenic acid on intestinal lipid absorption may be mediated, at least in part, by downregulation of PPARγ.

### 3.3 Chlorogenic acid promotes lipolysis in the adipose tissue of ob/ob mice

**Figure 3:**
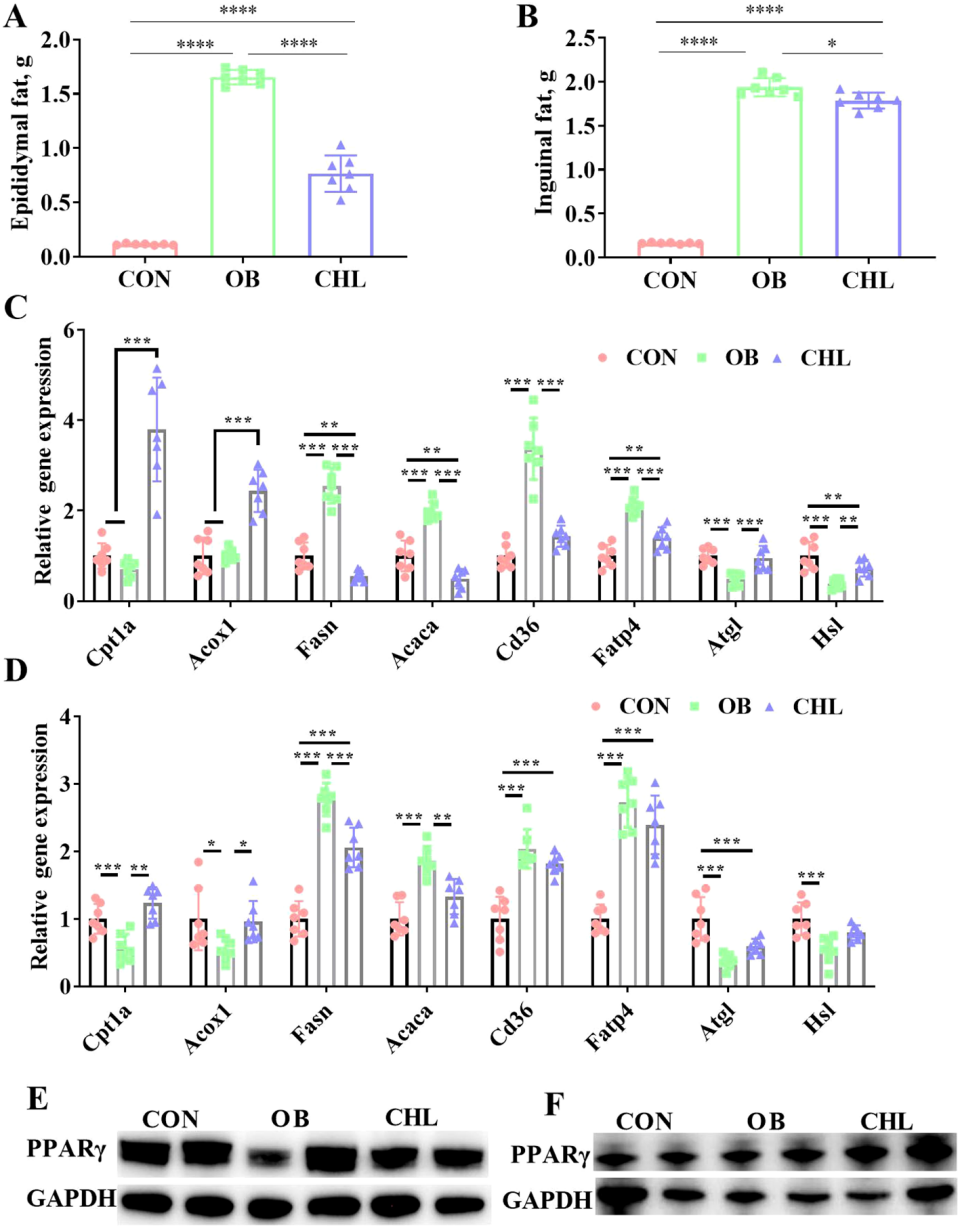
Chlorogenic acid promotes lipolysis in the adipose tissue of ob/ob mice. **(A)** Epididymal fat weight; **(B)** Inguinal fat weight; Relative gene expression in the epididymal **(C)** and inguinal fat **(D)**; PPARγ protein expression in the epididymal **(E)** and inguinal fat **(F)**. Data were expressed as mean ± SEM. n = 7. ^*^P<0.05, ^**^P<0.01, ^***^P<0.001, ^****^P<0.0001.

To further examine the effects of chlorogenic acid on lipid metabolism in adipose tissue, we first observed that the weights of both epididymal and inguinal fat were significantly reduced following chlorogenic acid treatment (**Figures 3A, B**). Gene expression analysis revealed that, in epididymal fat, chlorogenic acid significantly upregulated fatty acid oxidation–related genes (*Cpt1a* and *Acox1*) and lipolysis-related genes (*Atgl* and *Hsl*), while concurrently downregulating lipogenic genes (*Fasn* and *Acaca*) and lipid transport–related genes (*Cd36* and *Fatp4*) (**Figure 3C**). Meanwhile, in inguinal fat, chlorogenic acid significantly increased the expression of *Cpt1a* and *Acox1*, and decreased the expression of *Fasn* and *Acaca*, without affecting *Atgl*, *Hsl*, *Cd36*, or *Fatp4* (**Figure 3D**). Moreover, protein expression of PPARγ was not altered by chlorogenic acid in either adipose depot (**Figure 3E**). Together, these findings indicate that chlorogenic acid treatment is associated with changes in markers related to lipolysis and fatty acid oxidation in adipose tissue, particularly in epididymal fat.

### 3.4 Chlorogenic acid decreases lipid uptake in epithelium cells via targeting PPARγ

**Figure 4:**
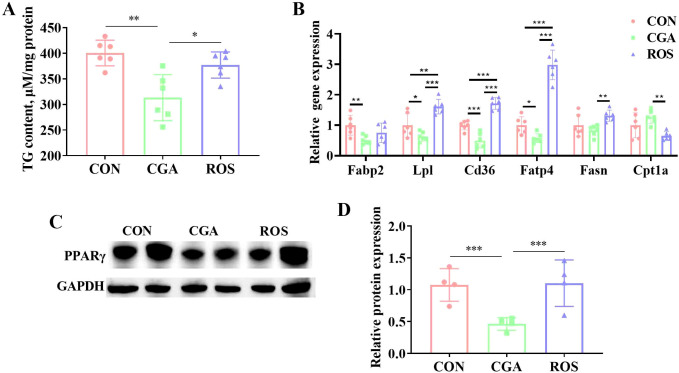
Chlorogenic acid decreases lipid uptake in epithelium cells via targeting PPARγ. **(A)** TG content in IPEC-J2 cells (n = 6); **(B)** Relative gene expression in IPEC-J2 cells (n = 6); **(C-D)** PPARγ protein expression in IPEC-J2 cells (n = 4). CON, control cells; CGA, cells treated with chlorogenic acid; ROS, cells treated with rosiglitazone. Data were expressed as mean ± SEM. ^*^P<0.05, ^**^P<0.01, ^***^P<0.001.

To determine whether PPARγ mediates the effects of chlorogenic acid, IPEC-J2 cells were treated with chlorogenic acid in the presence or absence of the PPARγ agonist rosiglitazone. Cellular TG analysis showed that chlorogenic acid significantly reduced TG content, whereas rosiglitazone attenuated this effect (**Figure 4A**). Consistently, chlorogenic acid downregulated the expression of lipid transport–related genes, including *Fabp2*, *Lpl*, *Cd36*, and *Fatp4*, without affecting the expression of the lipogenic gene *Fasn* or the fatty acid oxidation–related gene *Cpt1a* (**Figure 4B**). In contrast, rosiglitazone restored the expression of *Lpl*, *Cd36*, and *Fatp4*, while further increasing *Fasn* expression and decreasing *Cpt1a* expression (**Figure 4B**). Moreover, PPARγ protein levels were reduced by chlorogenic acid but upregulated by rosiglitazone (**Figures 4C, D**). Collectively, these results indicate that chlorogenic acid suppresses lipid uptake in intestinal epithelial cells potentially through inhibition of PPARγ signaling.

### 3.5 Chlorogenic acid decreases lipid uptake in intestinal organoids via targeting PPARγ

**Figure 5:**
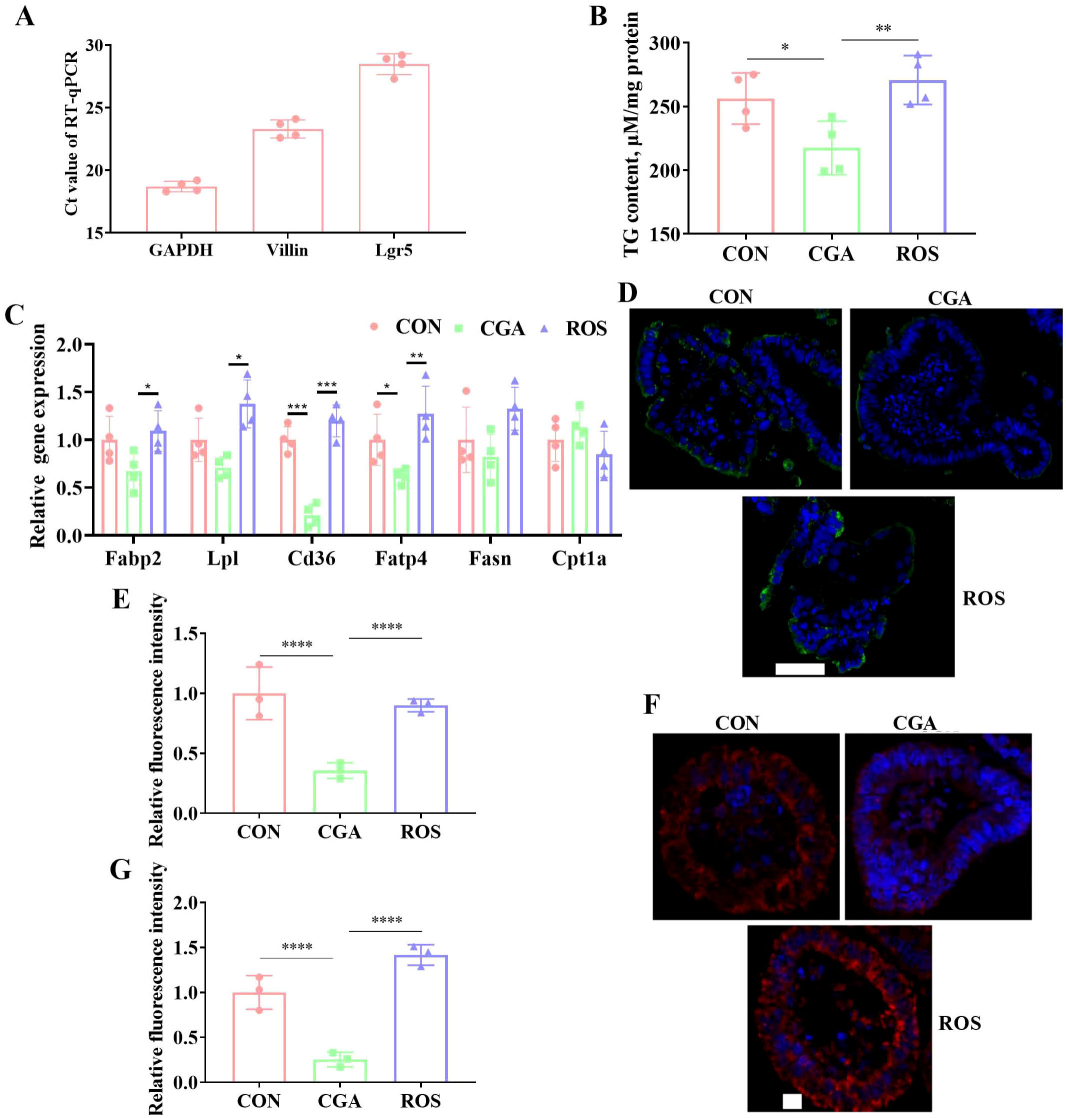
Chlorogenic acid decreases lipid uptake in intestinal organoids via targeting PPARγ. **(A)** Ct value of *Villin* and *Lgr5* gene by RT-qPCR; **(B)** TG content in intestinal organoids; **(C)** Relative gene expression in intestinal organoids; **(D)** CD36 protein expression in intestinal organoids (Green, CD36; Blue, DAPI; scale bar (white blank) = 50 μm); **(E)** Relative fluorescence intensity of CD36; **(F)** PPARγ protein expression in intestinal organoids (Red, PPARγ; Blue, DAPI; scale bar (white blank) = 10 μm); **(G)** Relative fluorescence intensity of PPARγ. Data were expressed as mean ± SEM. n = 4 or 3. ^*^P<0.05, ^**^P<0.01, ^***^P<0.001, ^****^P<0.0001.

To further confirm the effects of chlorogenic acid on intestinal lipid uptake, intestinal crypts were isolated and cultured to generate organoids. The Ct value of *Villin* and *Lgr5* gene by RT-qPCR indicated that the organoids maintained intestinal stem cell populations and differentiated epithelial characteristics (**Figure 5A**). The organoids were treated with chlorogenic acid in the presence or absence of rosiglitazone. Cellular TG analysis showed that chlorogenic acid significantly reduced TG content, whereas rosiglitazone attenuated this effect (**Figure 5B**). At the transcriptional level, chlorogenic acid reduced the expression of *Fabp2* and *Lpl*, although the changes did not reach statistical significance (**Figure 5C**). Notably, chlorogenic acid significantly downregulated the expression of *Cd36* and *Fatp4*, while rosiglitazone restored their expression (**Figure 5C**). In contrast, chlorogenic acid had no significant effect on the expression of *Fasn* and *Cpt1a* in intestinal organoids. Given that *Cd36* exhibited the most pronounced downregulation, its protein expression was further assessed and found to be significantly decreased by chlorogenic acid and restored by rosiglitazone (**Figures 5D, E**). Moreover, immunofluorescence staining revealed that PPARγ protein level was reduced following chlorogenic acid treatment but increased upon rosiglitazone supplementation (**Figures 5F, G**). Collectively, these results further support that chlorogenic acid suppresses intestinal lipid uptake through inhibition of PPARγ signaling.

### 3.6 Chlorogenic acid promotes adipose lipid catabolism via epithelium-derived exosomes in ob/ob mice

**Figure 6:**
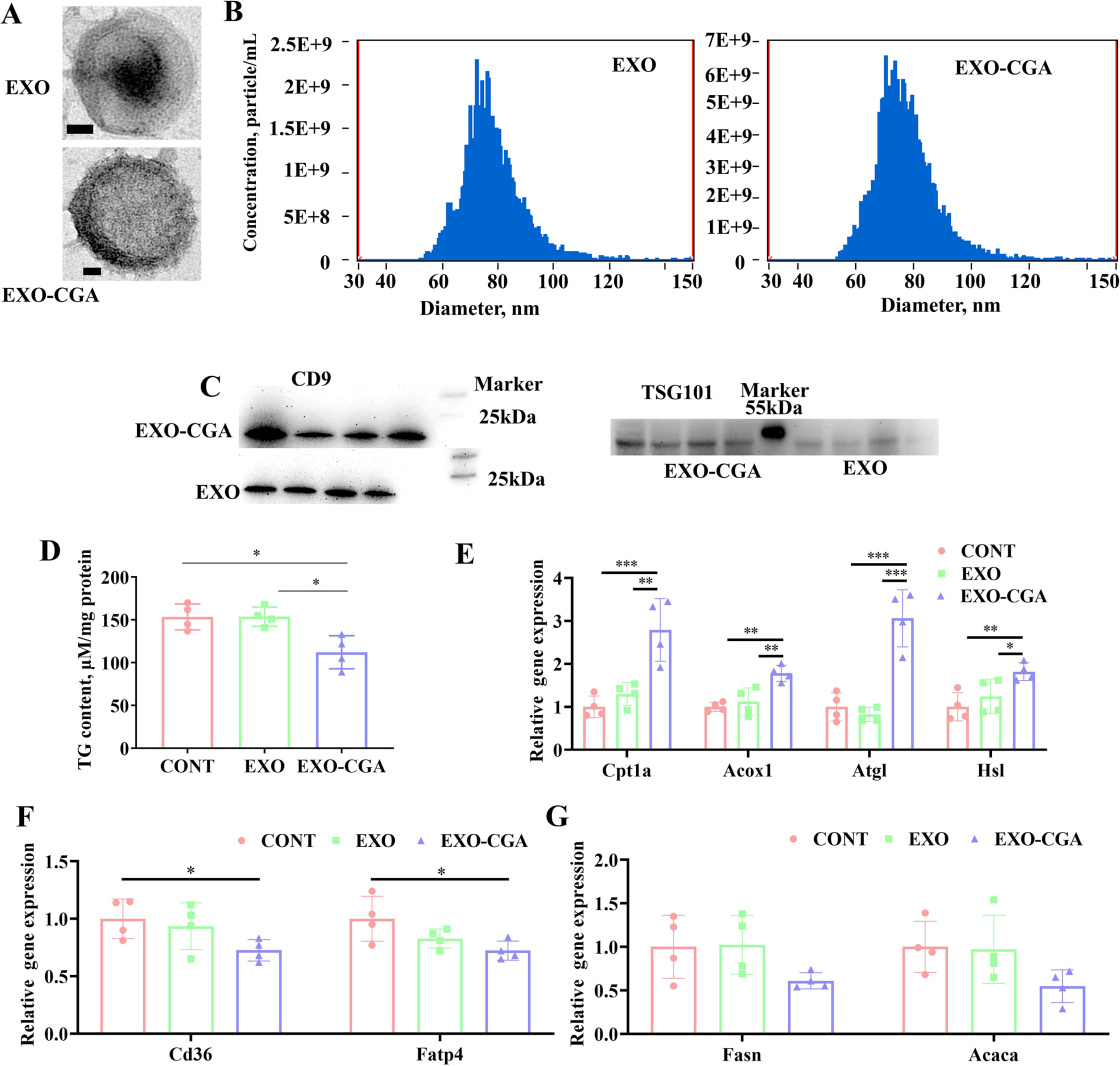
Chlorogenic acid promotes adipose lipid catabolism via epithelium-derived exosomes in ob/ob mice. **(A)** exosomes characterization by transmission electron microscopy; **(B)** exosomes characterization by nanoparticle tracking analysis; **(C)** CD9 and TSG101 expression in exosomes; **(D)** TG content in 3T3-L1 cells; Relative expression of genes involved in lipolysis **(E)**, lipid transportation **(F)** and lipogenesis **(G)**. CONT, control 3T3-L1 cells; EXO, 3T3-L1 cells treated with exosomes derived from IPEC-J2 cells; EXO-CGA, 3T3-L1 cells treated with exosomes derived from chlorogenic acid–treated IPEC-J2 cells. Data were expressed as mean ± SEM. n = 4. ^*^P<0.05, ^**^P<0.01, ^***^P<0.001.

To further investigate whether exosomes mediate the effects of chlorogenic acid on adipose lipid catabolism, IPEC-J2 cells were treated with chlorogenic acid, and epithelium-derived exosomes were subsequently isolated for treatment of 3T3-L1 cells. Transmission electron microscopy (TEM) revealed the characteristic cup-shaped morphology of exosomes derived from IPEC-J2 cells (**Figure 6A**). Nanoparticle tracking analysis (NTA) showed that the average diameter of exosomes was not significantly altered, while the concentration of exosomes increased from 7.32 × 10^10^ particles/mL to 2.36 × 10¹¹ particles/mL after chlorogenic acid treatment (**Figure 6B**). These results indicate that chlorogenic acid enhances the production of epithelium-derived exosomes. The identity of exosomes was further confirmed by detecting the expression of exosomal markers CD9 and TSG101 (**Figure 6C**). Functional analysis demonstrated that exosomes derived from chlorogenic acid–treated IPEC-J2 cells significantly reduced intracellular TG content in 3T3-L1 cells (**Figure 6D**). Moreover, these exosomes markedly upregulated the expression of *Cpt1a*, *Acox1*, *Atgl* and *Hsl* genes (**Figure 6E**), while downregulating *Cd36* and *Fatp4* genes (**Figure 6F**). In contrast, exosomes derived from untreated IPEC-J2 cells did not exert these effects. Notably, neither group of exosomes significantly affected the expression of *Fasn* and *Acaca* genes (**Figure 6G**). Collectively, these findings suggest that chlorogenic acid may enhance adipose lipid catabolism and support a potential role for epithelium-derived exosomes in mediating this effect.

## 4 Discussion

The effects of chlorogenic acid on lipid metabolism have been widely documented. It has been shown to lower triglyceride levels in both serum and liver, reduce low-density lipoprotein (LDL) oxidation susceptibility, and decrease circulating LDL-cholesterol levels (Meng et al., 2013). In addition, chlorogenic acid modulates lipid metabolism by regulating obesity-related hormones (Cho et al., 2010) and altering the levels of lipids, lipoproteins and key metabolic enzymes (Karthikesan et al., 2010). Notably, its regulatory effects are tissue-specific. Previous studies have primarily focused on its ability to inhibit lipid absorption and promote lipolysis in the liver (Ziolkiewicz et al., 2024; Tian et al., 2025). Beyond this, chlorogenic acid enhances thermogenesis in brown adipocytes (Han et al., 2019), reduces lipid accumulation in the kidney (Yang et al., 2024), and suppresses intestinal cholesterol absorption by inhibiting cholesterol micelle formation and pancreatic lipase activity (Crozier et al., 2012). However, whether chlorogenic acid directly regulates genes involved in intestinal lipid metabolism remains largely unexplored, despite the intestine being the primary site of exposure following oral administration.

Postprandial hyperlipidemia is a hallmark of diabetic dyslipidemia and a major contributor to cardiovascular risk in individuals with obesity or type 2 diabetes (Verges, 2022), underscoring the critical role of intestinal lipid absorption and metabolism. Fatty acid uptake and transport across the apical membrane of enterocytes are predominantly protein-mediated processes. Among these, CD36 plays a central role in fatty acid uptake (Abumrad, 2005), while fatty acid transport proteins (FATPs) facilitate membrane transport (Schaffer and Lodish, 1994), and intracellular trafficking is mediated by fatty acid-binding proteins (FABPs) (Hanhoff et al., 2002). In parallel, metabolic adaptation within enterocytes, such as enhanced fatty acid oxidation via increased CPT1A expression and reduced lipid synthesis via decreased FASN expression, contributes to the regulation of circulating lipid levels (Ko et al., 2020). In the present study, chlorogenic acid suppressed the expression of *Fabp2*, *Lpl*, *Cd36* and *Fatp4*, accompanied by reduced triglyceride content in the jejunum and ileum. Moreover, chlorogenic acid downregulated intestinal PPARγ, a key regulator of lipid metabolism, suggesting that PPARγ signaling may represent a central mechanism underlying its lipid-lowering effects in the intestine.

We previously demonstrated that chlorogenic acid alleviates intestinal inflammation and oxidative stress (Yan et al., 2020a; Yan et al., 2022). Whether it indirectly regulates intestinal lipid metabolism through these effects, however, remains unclear, given that both inflammation and oxidative stress can influence lipid transport (Emami et al., 2020; He et al., 2025a; Liang et al., 2026; Wu et al., 2026). In addition, chlorogenic acid has been shown to improve gut microbiota composition and repair mucosal damage by reducing microbiota-derived lipopolysaccharide (Yan et al., 2020a; Yan et al., 2022). This suggests that it may also modulate lipid metabolism through regulation of the gut microbiota, which is closely associated with intestinal lipid transport (Shelton et al., 2023; Wang et al., 2023; Huang et al., 2025; Ye et al., 2026). Notably, our findings further indicate that chlorogenic acid can directly promote lipid transport in the intestine *in vivo*, implying that the gut microbiota is not indispensable for its regulatory effects on intestinal lipid metabolism.

Previous pharmacokinetic study have demonstrated that orally administered chlorogenic acid can be absorbed and widely distributed across tissues, with the liver showing the highest accumulation (Zhou et al., 2014). However, its systemic bioavailability is relatively low and largely depends on metabolism by the gut microbiota, with most circulating compounds present as metabolites rather than the parent form (Gonthier et al., 2003), thereby limiting its ability to directly reach peripheral tissues and exert biological effects. Given that we previously found chlorogenic acid to promote the expression of intestinal microRNAs (Yan et al., 2024), the present study further investigated whether it could modulate adipose tissue metabolism via exosomes. Previous studies have shown that chlorogenic acid can indirectly alter the structure and protein composition of gut microbe–derived exosomes (Zheng et al., 2023). Consistently, our results indicate that chlorogenic acid can affect exosome concentration. However, the specific exosomal components regulated by chlorogenic acid that contribute to lipid metabolism in adipose tissue remain to be elucidated. Moreover, we observed that exosomes derived from intestinal epithelial cells treated with chlorogenic acid promoted lipid catabolism in adipocytes *in vitro*. Notably, it has been reported that intestinal epithelial cell–derived exosomes can target adipose tissue and enhance thermogenesis (Han et al., 2025), suggesting that intestine-derived exosomes may reach adipose tissue through the circulatory system.

This study has several limitations. Although exosomes derived from chlorogenic acid-treated IPEC-J2 cells were shown to modulate lipid metabolism in 3T3-L1 adipocytes *in vitro*, the present study did not directly investigate whether exosomes released from the intestine following chlorogenic acid administration enter the circulation and are subsequently taken up by adipose tissue. Furthermore, reliable discrimination of intestine-derived exosomes from exosomes originating from other tissues remains technically challenging *in vivo*. Future studies employing tissue-specific exosome labeling or lineage-tracing approaches, together with analyses of circulating exosomal markers and cargos in adipose tissue, will be necessary to further establish the existence and physiological relevance of intestine–adipose exosome-mediated communication. Additionally, although intracellular TG accumulation and the expression of lipid transport-related proteins were determined, direct assays assessing lipid uptake were not conducted. Future studies incorporating functional lipid uptake analyses are needed to further substantiate the role of exosomes in regulating intestinal lipid absorption. Furthermore, the vesicles isolated by ultracentrifugation were confirmed to be exosome-enriched preparations based on their morphology, particle size distribution, and expression of exosomal marker proteins. However, ultracentrifugation may also precipitate non-exosomal components, including soluble proteins and lipid-associated complexes. Consequently, although our results support the involvement of intestine-derived exosomes in mediating the metabolic effects of chlorogenic acid, additional studies using exosome depletion, density gradient purification, or pharmacological inhibition of exosome secretion are needed to unequivocally determine whether exosomes are the major functional mediators.

In conclusion, our findings suggest that chlorogenic acid may exert anti-obesity effects through coordinated regulation of intestinal and adipose tissues. Chlorogenic acid treatment was associated with reduced intestinal lipid accumulation, which may contribute to limiting systemic lipid influx, and with enhanced lipid catabolism in adipose tissue. The effects observed in intestinal epithelial cells appear to involve modulation of PPARγ signaling. In addition, our results support a potential role for exosome-mediated inter-organ communication, whereby chlorogenic acid may stimulate the release of intestine-derived exosomes that contribute to the regulation of adipose lipid metabolism. Although further studies are required to directly confirm the tissue specificity and causal role of these exosomes, the present findings provide preliminary evidence for a possible intestine–adipose communication axis involved in the metabolic actions of chlorogenic acid and warrant further investigation into its role in lipid homeostasis.

## Data Availability

The raw data supporting the conclusions of this article will be made available by the authors, without undue reservation.
