# Supplementary material for: Chlorogenic acid inhibits intestinal lipid uptake and promotes adipose lipid catabolism via epithelium-derived exosomes in ob/ob mice
Source: Front Physiol. 2026 Jul 29;17:1896020. doi: 10.3389/fphys.2026.1896020 (PMC13461397; doi:10.3389/fphys.2026.1896020)
Supplement: Supplementary file 1 [file Table1.docx]

**Supplementary Table 1 Primer sequences for RT-qPCR analysis of gene expression**

| Gene | 5’-3’ Primer sequence | Gene | | 5’-3’ Primer sequence |
| --- | --- | --- | --- | --- |
| *Cpt1a* | F: gactccgctcgctcattcc | | *Acox1* | F: gtctccgtcatgaatcccga |
|  | R: ttgagggcttcatggctcag | |  | R: ggtgcacagagtttttaaacca |
| *Fasn* | F: cccctcacatcaagtgggac | | *Acaca* | F: ggaggcggatatctgctgag |
|  | R: ttgggaagtcctcagcaacc | |  | R: gtatctgagctgacggaggc |
| *Cd36* | F: gagccatctttgagccttca | | *Fatp4* | F: gattcagagccctagcgtcc |
|  | R: tcagatccgaacacagcgta | |  | R: ctgctagctttggtctggca |
| *Fabp2* | F: tgctgtccgagaggtttctg | | *Lpl* | F: ttgcagagagaggactcgga |
|  | R: agaatcgcttggcctcaact | |  | R: agatgaatggagcgctcgtg |
| *Atgl* | F: accttcgcaatctctaccgc | | *Hsl* | F: tgttggggtgactctaacgc |
|  | R: agcaaagggttgggttggtt | |  | R: gagctccgcctttaatgggt |
| *β-actin* | F: tgtccaccttccagcagatgt | |  |  |
|  | R: agctcagtaacagtccgcctaga | |  |  |
